# Self-assembling nanoparticles encapsulating zoledronic acid inhibit mesenchymal stromal cells differentiation, migration and secretion of proangiogenic factors and their interactions with prostate cancer cells

**DOI:** 10.18632/oncotarget.17216

**Published:** 2017-04-19

**Authors:** Cinzia Borghese, Naike Casagrande, Eliana Pivetta, Alfonso Colombatti, Mariarosaria Boccellino, Evzen Amler, Nicola Normanno, Michele Caraglia, Giuseppe De Rosa, Donatella Aldinucci

**Affiliations:** ^1^ Molecular Oncology Unit, Centro di Riferimento Oncologico, IRCCS-National Cancer Institute, Aviano, PN, Italy; ^2^ Department of Biochemistry, Biophysics and General Pathology, Second University of Naples, Naples, Italy; ^3^ Sbarro Institute for Cancer Research and Molecular Medicine, Center for Biotechnology, College of Science and Technology, Temple University, Philadelphia, PA, USA; ^4^ Indoor Environmental Quality, University Center for Energy Efficient Buildings, Czech Technical University in Prague, Buštěhrad, Czech Republic; ^5^ Laboratory of Tissue Engineering, Institute of Experimental Medicine, Czech Academy of Sciences, Prague, Czech Republic; ^6^ Cell Biology & Biotherapy Unit, Istituto Nazionale Tumori “Fondazione G Pascale”-IRCCS, Naples, Italy; ^7^ Department of Pharmacy, Federico II University of Naples, Naples, Italy

**Keywords:** zoledronic acid, self-assembling nanoparticles, mesenchymal stromal cells, prostate cancer, tumor microenvironment

## Abstract

Zoledronic Acid (ZA) rapidly concentrates into the bone and reduces skeletal-related events and pain in bone metastatic prostate cancer (PCa), but exerts only a limited or absent impact as anti-cancer activity. Recently, we developed self-assembling nanoparticles (NPS) encapsulating zoledronic acid (NZ) that allowed a higher intratumor delivery of the drug compared with free zoledronic acid (ZA) in *in vivo* cancer models of PCa. Increasing evidence suggests that Bone Marrow (BM) Mesenchymal stromal cells (BM-MSCs) are recruited into the stroma of developing tumors where they contribute to progression by enhancing tumor growth and metastasis.

We demonstrated that treatment with NZ decreased migration and differentiation into adipocytes and osteoblasts of MSCs and inhibited osteoclastogenesis. Treatment with NZ reduced the capability of MSCs to promote the migration and the clonogenic growth of the prostate cancer cell lines PC3 and DU145. The levels of Interleukin-6 and of the pro-angiogenic factors VEGF and FGF-2 were significantly reduced in MSC-CM derived from MSCs treated with NZ, and CCL5 secretion was almost totally abolished. Moreover, treatment of MSCs with supernatants from PC3 cells, leading to tumor-educated MSCs (TE-MSCs), increased the secretion of IL-6, CCL5, VEGF and FGF-2 by MSCs and increased their capability to increase PC3 cells clonogenic growth. Treatment with NZ decreased cytokine secretion and the pro-tumorigenic effects also of TE-MSCS. In conclusion, demonstrating that NZ is capable to inhibit the cross talk between MSCs and PCa, this study provides a novel insight to explain the powerful anticancer activity of NZ on PCa.

## INTRODUCTION

MSCs are pluripotent progenitor cells that contribute to the maintenance and regeneration of a variety of connective tissues, including bone and adipose tissue [1–4]. MSCs recruited and then “educated” by tumor cells, through direct and/or indirect interaction with tumor cells, can support tumor growth, promote angiogenesis, inhibit the development of an efficient anti-tumor response, induce drug resistance, invasion and even metastases [1–7]. MSCs participate in prostate cancer (PCa) carcinogenesis and growth [8] and are recruited and activated into Carcinoma-Associated Fibroblasts (CAF) by molecules secreted by the Tumor Microenvironment (TME)[9]. Thus, disrupting the interactions between PCa cells and MSCs could represent a new therapeutic strategy [10].

Zoledronic acid (ZA) is a biphosphonate used for many years to reduce skeletal complications related to the benign and malignant bone diseases characterized by enhanced osteoclastic bone resorption [11]. ZA exerts biological activity on MSCs differentiation [12] and inhibits the secretion by MSCs of cytokines involved in breast cancer cell migration [13] and in monocytes recruitment [14]. In PCa cellular models ZA impairs PCa-induced M2-macrophages polarization and prevents the M2 macrophages-mediated activation of normal fibroblasts [15].

Although ZA inhibits proliferation and migration of cancer cells and induces apoptosis *in vitro* [16], it has negligible effects on different tumors *in vivo*, due to fast uptake by bone tissue that limits the amount of the drug reaching the extra-skeletal tumor niches as well as to its rapid elimination from plasma upon intravenous administration due to renal excretion [17].

Nanotechnology has been used to improve the therapeutic index of new or established drugs by modifying drug absorption, prolonging biological half-life and reducing toxicity [18–20]. Recently, to increase ZA plasma half-life and accumulation into tumors, new self-assembling PEGylated nanoparticles (NPs) encapsulating ZA (NZ) was developed, transforming this biphosphonate in a powerful anti-cancer agent [21, 22].

NZ offers good technological characteristics, in terms of homogeneous size distribution and high drug loading and a strong increase of ZA anti-cancer activity *in vivo* [21, 22]. NZ reverts multidrug resistance in lung cancer [23] and its combination with doxorubicin overcomes simultaneously chemo-resistance and immune-resistance in breast cancer [24] thus suggesting its future clinical development as anticancer agent [22]. In Prostate cancer (PCa) models, NZ induces the complete remission of tumor xenografts with low toxicity, reduces tumor-associated macrophages [22] and inhibits angiogenesis [22].

Therefore, ZA and especially NZ may represent a potential therapeutic approach for breast and PCa, since it is potentially able to decrease the supportive role of TME and in particular of MSCs.

Here, we have compared the functional effects of free ZA and NZ on osteoblastic and adipocytic differentiation of MSCs, on osteoclast differentiation of monocytes and on the capability of MSCs- conditioned medium to promote the migration and proliferation of PCa cells.

## RESULTS

### Characteristics of self-assembling nanoparticles

PEGylated ZOL-containing NPs were prepared by mixing CaPZ NPs (final ZOL concentration 0,125 mM) with DOTAP/chol/DSPEG2000 cationic liposomes. The resulting self-assembling NPs had a mean diameter of about 147 nm with polydispersity index < 0.2. According to previously published papers [21] the nanoparticles had a positive zeta potential, of about 18 mV.

### Effects of NZ on MSCs viability and migration

**Figure 1:**
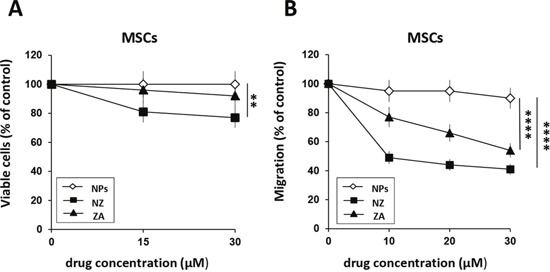
Effect of NZ and ZA on MSCs proliferation and migration **(A)** MSCs (2×10^3^) were cultured in the presence of increasing amounts of NZ, ZA or blank NPs (free liposomes). After 72 h, viable cells were evaluated by the MTT assay. **(B)** Migration through a collagen type I-coated Boyden chamber of MSCs (20 h) untreated and treated for 72 h with NZ, ZA or NPs (10, 20, 30 μM) in response to DMEM complete medium (10% FBS). Results are presented as percentage of migrated cells relative to control (untreated cells=100%). Values represent the mean ± SD of N=3 independent experiments.

ZA was shown to significantly affect MSCs migration whereas it has a slight effect on proliferation [13]. We treated MSCs with increasing concentrations of NZ and for comparative purposes with ZA. Thereafter, we evaluated proliferation and migration of MSCs. Free ZA did not significantly affect MSCs growth and NZ only slightly decreased viable cells (about 20% of inhibition at the highest drug concentration)(Figure 1A). However, treatment with NZ decreased in a dose dependent manner MSCs migration and, at the low concentrations, it was more active than ZA (Figure 1B). Blank NPs did not significantly affect MSCs proliferation or migration.

### Effects of NZ on osteoblast, adipocyte and osteoclast differentiation

We next evaluated the effects of NZ or ZA on osteoblastic (OB) and adipocytic (AD) differentiation in MSCs and on osteoclast (OC) differentiation in monocytes.

**Figure 2:**
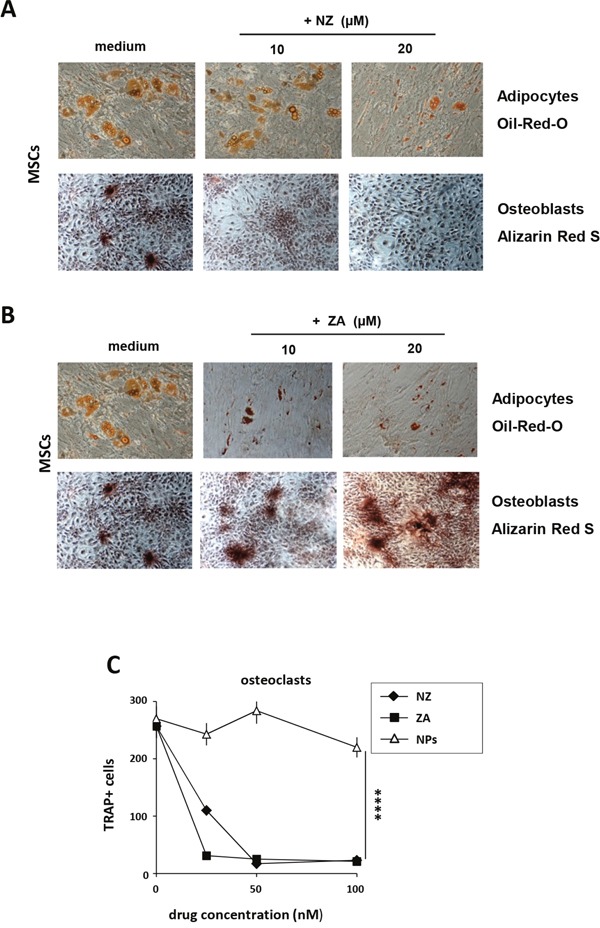
Effect of NZ and ZA on osteoblast, adipocyte and osteoclast differentiation Phase contrast microphotographs showing MSCs differentiation. MSCs were cultured with osteogenic (upper panel) or adipogenic (lower panel) medium alone or in the presence of **(A)** NZ or **(B)** ZA. Osteogenic differentiation was evaluated with Alizarin red staining (lower panel), adipogenic differentiation with Oil-Red-O staining (upper panel) (original magnification, 10×0.25). One representative experiment of three was reported. **(C)** Effect of NZ and ZA on osteoclastogenesis. Pre-OCs were incubated for 14 days with RANKL +M-CSF in the absence or in the presence of increasing concentrations of NZ, ZA or NPs. Then cells were stained for TRAP. Results represent the number of multinucleated TRAP-positive cells.

MSCs were treated with NZ or ZA using a pulse treatment (high drug concentration for a short time). Treatment with NZ (Figure 2A) and especially with ZA (Figure 2B) inhibited AD differentiation (Figure 2A and 2B, upper panel) (Oil-red-O staining). In agreement with Ebert et al. [12], free ZA increased OB differentiation (Figure 2B, lower panel). Conversely, NZ decreased MSCs differentiation into OB (Alizarin red staining) (Figure 2A, lower panel). Blank nanoparticles (NPs, control) never affected OB or AD differentiation (data not shown).

ZA blocks pathologic bone resorption by inhibiting OC function and then by inducing apoptosis [1, 26]. Consistently, we found that also treatment with NZ, as with free ZA, decreased in a dose dependent manner osteoclast differentiation of monocytes (Figure 2C).

### Effects of NZ on prostate and breast cancer cells migration induced by MSCs-CM

MSCs increase the motility of PCa [25] and breast [13] cancer cells. We found that CM from MSCs increased of about 4-folds the migration of PC3, DU145 and MCF-7 cells. CM from ZA-treated MSCs showed a reduced capacity to promote the migration of MCF-7 breast cancer cells [13].

**Figure 3:**
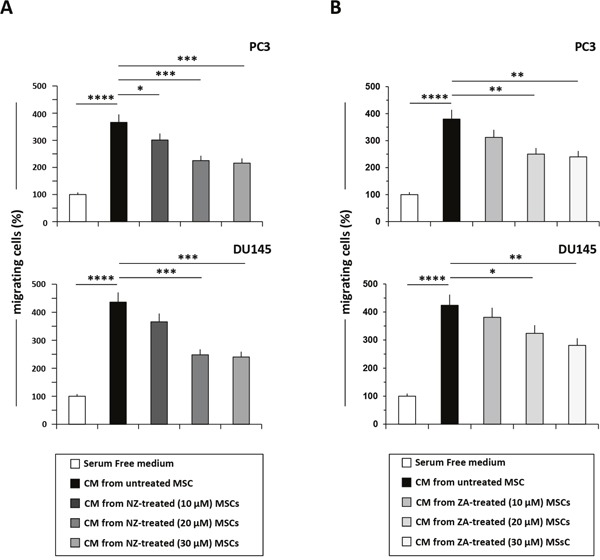
Treatment of MSCs with NZ or ZA decreased the migration of prostate cancer cells induced by MSCs-CM Migration of PC3 and DU145 cells through a fibronectin-coated Boyden chamber in response to serum free medium (control=100%), CM from MSCs untreated or treated with **(A)** NZ or **(B)** ZA. Histograms represent the percentage of transmigrated cells after 20 h relative to control (cells migrated towards serum free medium). Values represent the mean ± SD of N=3 independent experiments.

We found that also NZ decreased in a dose-dependent manner the ability of MSCs-CM to promote the migration of PC3 and DU145 cells (Figure 3A); the same effect was observed when MSCs were treated with ZA (Figure 3B). The treatment of MSCs with NZ, as well as with ZA, reduced the migration of MCF-7 breast cancer cells in response to MSCs-CM and was active [13] even at the lowest (10 μM) drug concentrations (Supplementary Figure 1A).

### Effects of NZ on cytokines/chemokines secretion by MSCs

**Figure 4:**
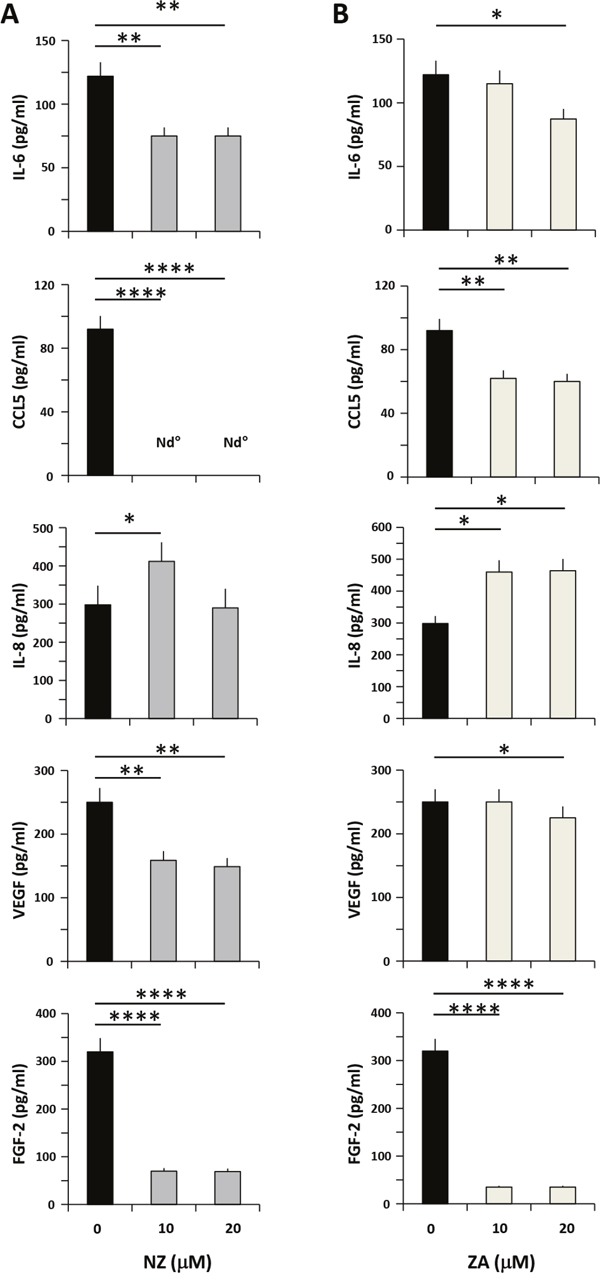
Effects of NZ and ZA on cytokine/chemokine secretion by MSCs MSCs were cultured in medium alone or with **(A)** NZ or **(B)** ZA for 72 h, then washed and incubated for additional 24 h in serum free medium. CM from MSCs was analyzed for IL-6, CCL5, IL-8, VEGF and FGF-2 secretion using specific ELISA assays. All samples were run in duplicate; supernatants from three different experiments were evaluated.

To investigate the potential mechanism responsible for the downstream effects of NZ, we measured the levels of cytokines/chemokines secreted by MSCs and known to be involved in prostate cancer progression. We treated MSCs with increasing amounts of NZ or ZA, and then we quantified the secretion of IL-6, CCL5, IL-8, VEGF and FGF-2. Treatment of MSCs with NZ (10-20 μM) decreased IL-6 secretion of about 40% (Figure 4A) and almost totally abolished the secretion of CCL5. Only at the lowest NZ concentration (10 μM) a slight increase of IL-8 was observed (Figure 4A). The secretion of the pro-angiogenic factors VEGF (about 40%) and especially of FGF-2 (about 80%) (Figure 4A) was significantly decreased following treatment with NZ. ZA treatment of MSCs, as previously reported [13], used at the same concentrations, increased IL-8 but was less active than NZ in decreasing IL-6, VEGF and CCL5 levels (Figure 4B).

### Effects of NZ on the clonogenic growth induced by MSCs-CM

**Figure 5:**
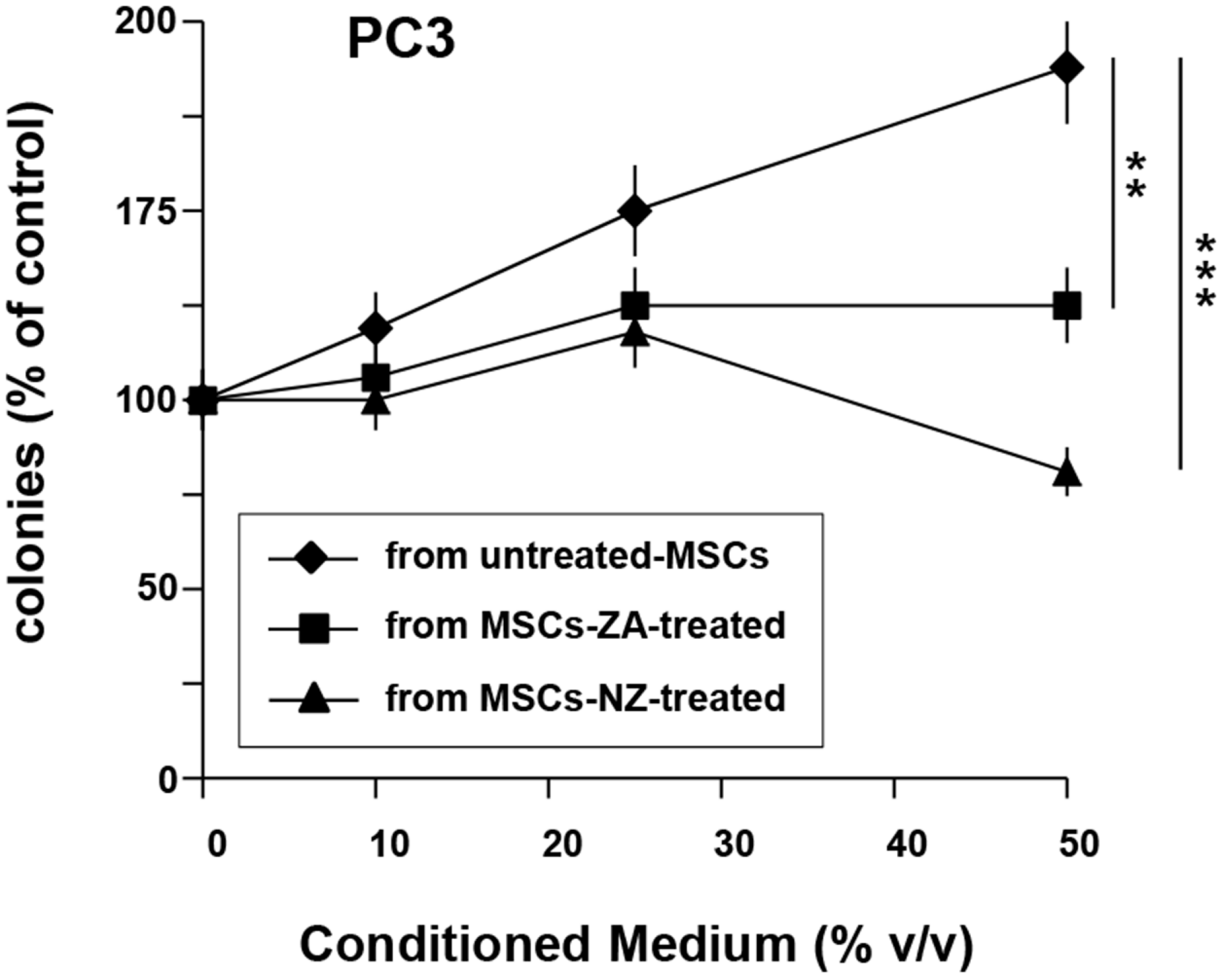
Treatment of MSCs with NZ or ZA decreased the clonogenic growth of PC3 cells induced by MSCs-CM 100 PC3 cells were plated in 24-well flat-bottomed plates and allowed to adhere for 24 h, then cultured in the presence of increased concentrations of supernatants from MSCs untreated or treated with ZA or NZ (20 μM). After 7 days, plates were observed under phase-contrast microscopy and colonies counted. Values represent the mean ± SD of N=3 independent experiments.

We have previously demonstrated that MSCs-CM increased the clonogenic growth of PCa cells [5]. Thus, we evaluated the effects of NZ as well as free ZA on the ability of MSCs-CM to support the clonogenic growth of prostate cancer cells (Figure 5). MSCs-CM increased in a dose-dependent manner the number of colonies formed by PC3 cells (Figure 5). Treatment of MSCs with free ZA and especially with NZ inhibited the capability of MSCs-CM to increase the clonogenic capacity of PC3 cells (Figure 5).

### Effects of NZ on tumor-educated MSCs (TE-MSCs)

**Figure 6:**
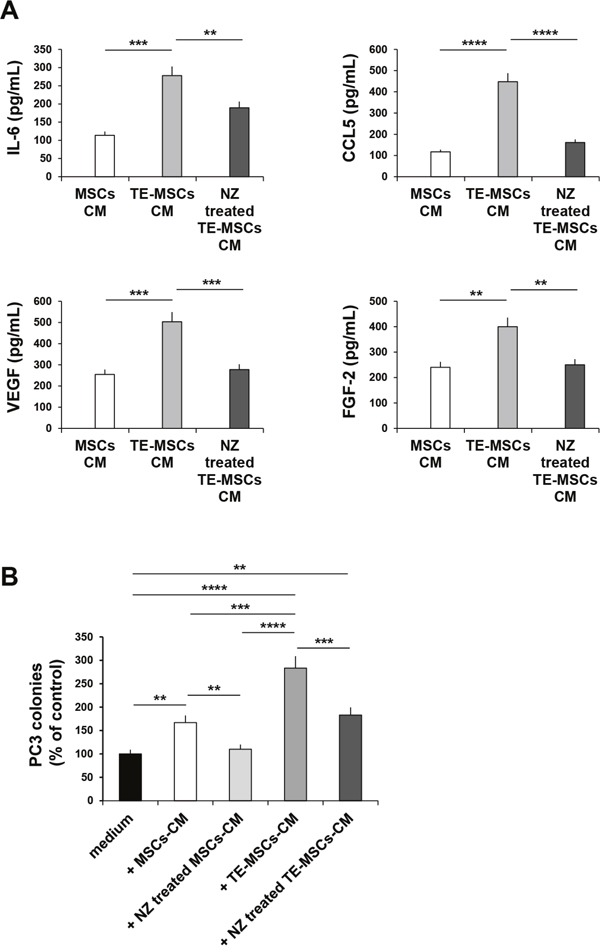
Effects of NZ on TE-MSCs **(A)** BM-MSCs were cultured for 9 days with 10% of PC3-CM (MSCs become TE-MSCs). MSCs and TE-MSCs were cultured in medium alone or with NZ (10μM) for 72 h, then washed and incubated for additional 24 h in serum free medium. CM from MSCs and TE-MSCs treated or not with NZ were analyzed for IL-6, CCL5, VEGF and FGF-2 secretion using specific ELISA assays. All samples were run in duplicate; supernatants from three different experiments were evaluated. **(B)** 100 PC3 cells were plated in 24-well flat-bottomed plates and allowed to adhere for 24 h, then cultured in the presence of 20% CM from MSCs and TE-MSCs treated or not with NZ (10 μM). After 7 days plates were observed under phase-contrast microscopy and colonies counted. Values represent the mean ± SD of N=3 independent experiments.

To survive and proliferate cancer cells not only recruit but also shape or “educate” normal cells [26]. We found that treatment or “education” of MSCs (TE-MSCs) with PC3 cells-CM increased the secretion of IL-6, CCL5, VEGF and FGF-2 by MSCs (Figure 6A). Treatment of TE-MSCs with NZ decreased the secretion of IL-6, CCL5, VEGF and FGF-2.

The cultivation of MSCs with PC3-CM (TE-MSCs) enhanced the capability of MSCs-CM to increase the clonogenic growth of PC3 cells (Figure 6B). Treatment of TE-MSCs with NZ inhibited the capability of TE-MSCs-CM to increase the clonogenic growth of PC3 cells (Figure 6B).

## DISCUSSION

MSCs migrate towards regenerating tissues, primary tumors and metastatic sites where they interact with tumor cells and sustain progression through the release of growth factors that promote neo-angiogenesis, immunosuppression, tumor cell migration and chemo/radiotherapy resistance [4, 6, 26–30]. Thus, drugs capable of disrupting microenvironmental interactions mediated by MSCs may represent a valid approach to treat human cancer [4, 26], including PCa.

In this study, we demonstrated that NZ, new self-assembling nanoparticles encapsulating ZA, were able to induce the following effects: i) decreased MSCs migration; ii) inhibited adipocyte, osteoblast and osteoclast differentiation; iii) decreased the secretion of chemokines and angiogenic factors by MSCs; iv) decreased the capability of CM from MSCs to potentiate clonogenic growth and migration of PCa cells.

In advanced stage PCa patients are often diagnosed with bone metastases. PCa cells prevalently cause osteoblastic lesions, characterized by an excess of bone formation but in many patients, osteoblastic and osteolytic metastatic lesions coexist [31–34]. Therefore, the ability of NZ to decrease osteoclast as well as osteoblastic differentiation could potentially counteract both bone destruction mediated by osteoclasts and the formation of the osteoblastic niches.

Adipocytes from AT or BM [31, 32, 35–38] can promote cancer progression in PCa and in breast cancer, suggesting this cell type as a new potential target for therapeutic intervention [39–41]. In our study, ZA as well as NZ inhibited adipocytic differentiation of MSCs but NZ, able to accumulate not only in the bone but also in the extra skeletal tissues, may represent a better therapeutic option respect to ZA. This is in line with the fact that clinical trials have shown that ZA treatment is effective in reducing skeletal-related events and bone pain in bone metastatic PCa cancer, but exerts only a limited or no impact on survival [42], likely because it rapidly accumulates into the bone but reaches only low intra-tumor concentrations.

On the other hand, NZ exerts its primary anticancer activity by killing cancer cells [22] and may inhibit the interactions of tumor cells with MSCs not only in the bone as ZA, but also in the primary tumor and in extra skeletal metastasis. It is worthy of note that in this study NZ was more effective than ZA also *in vitro*, suggesting that the use of self-assembling NPs can represent an advantage not only to accumulate ZA in tumor tissue, but also to enhance the ZA uptake into the target cells, as previously reported [21, 22, 43], or possibly to reduce the risk of side effects, such as osteonecrosis of the jaws [21, 44]. At the moment the exact mechanism for which NZ is more active than ZA is unknown. However, the authors hypothesize that NZ can enhance the uptake of ZA into the cells. Other authors demonstrate that ZA enter into the cells by dynamin dependent endocytosis; the same authors found that the intracellular uptake was significantly enhanced in the presence of calcium or following ZA encapsulation into the liposomes. The self-assembling developed in this work are based on calcium salt and cationic lipids, the latter well known as transfection agents for polyanionic molecules such as nucleic acids. Thus, considering their composition (calcium and cationic lipids), it is reasonable to hypothesize that NZ could significantly increase the intracellular uptake of encapsulated ZA, compared to naked ZA.

PCa cells, by producing large amounts of cytokines/chemokines, can attract inflammatory cells and MSCs in the TME. In turn, tumor-educated MSCs can recruit and then interact with immune cells [12, 48], can increase PCa cell motility and promote metastasis [27, 49] or exert protective effects against chemotherapy [14, 50]. Thus, NZ by decreasing the capability of MSCs to recruit PCa and breast cancer cells might decrease the formation not only of bone metastasis as free ZA, but also of extra-skeletal metastasis.

High levels of both IL-6 and of CCL5 correlate with bad prognosis and an advanced metastatic stage in prostate and breast cancer [45, 46]. PCa cells express a functional CCR5 receptor [47] and CCL5 signals from infiltrating MSCs increase PCa stem cell population and PCa metastasis [8, 48]. Breast cancer cells express CCR5 receptor which controls survival, invasiveness and metastatic ability [49]. Thus, NZ, by reducing IL-6 s and completely abolishing CCL5 secretion by MSCs may counteract the consequences of MSCs/PCa [8, 13, 25, 46, 50, 51] and MSCs/breast cancer [49, 52, 53] interactions. Furthermore, decreasing chemokines secretion by MSCs may reduce the recruitment of monocytes in the TME [54].

Given that angiogenesis is necessary to sustain tumor growth and cancer progression, development and/or discovery of new antitumor agents capable of inhibiting both tumor growth and angiogenesis is of paramount importance [55]. Given that MSCs of the tumor microenvironment produce large amounts of pro-angiogenic factors to generate the new vessels needed for cancer cells growth [3, 56], the inhibition of their secretion may represent an additional therapeutic strategy [56]. We found that NZ decreased the release by MSCs, and also by TE-MSCs, not only of chemokines but also of the pro-angiogenic factors VEGF and FGF-2. The observed down-modulation of IL-6, CCL5, VEGF and FGF by ZA and NZ was specific since no reduction of IL-8 was observed.

MSCs recruited by tumor cells seems to contribute not only to TME formation but also to tumor growth [4]. Recently, we found that CM from MSCs and especially from TE-MSCS increased PC3 cell colony growth [5]. Our data, showing that MSCs-CM from NZ and ZA treated cells resulted less active in increasing the clonogenic growth of PC3 cells, suggested that ZA and especially NZ may decrease the protumorigenic effects of MSCs.

## MATERIALS AND METHODS

### Drugs

Free ZA, used for comparative purposes, was from Novartis Pharma AG (Basel, Switzerland). Self-assembling nanoparticles encapsulating Zoledronic acid (NZ) were prepared and characterized as previously reported [21]. Blank self-assembling nanoparticles were used as control.

### Cell lines and culture conditions

Human Bone Marrow-MSCs (Lonza, Verviers, Belgium) were maintained in MSGM bullet kit (Lonza). They are positive for CD29, CD44, CD105, and CD166 and negative for markers of the hematopoietic lineage, such as CD14, CD34, and CD45. The human PCa-derived PC3 and DU145 cell lines (DSMZ, Braunschweig, Germany) were maintained in RPMI medium (Sigma-Aldrich, Milano, Italy) supplemented with 10% heat-inactivated fetal bovine serum (FBS; Sigma-Aldrich), 0.2 mg/ml penicillin/streptomycin and 0.1% (w/v) L-glutamine (Sigma-Aldrich) at 37°C in a 5% CO_2_ fully humidified atmosphere. The human breast cancer cell line MCF-7 (HTB-22) (ATCC, Rockville, MD, USA) was maintained in DMEM medium (Sigma-Aldrich) supplemented with 10% FBS; penicillin/streptomycin, L-glutamine, 1mM sodium piruvate (Gibco, Italy) and 1% MEM non-essential amino acid solution (Sigma-Aldrich).

### Generation of tumor educated-MSCs (TE-MSCs) and preparation of conditioned medium from BM-MSCs and TE-MSCs

To generate TE-MSCs, MSCs were cultured for 9 days in the presence of 10% of PC3-CM (3 additions). To obtain CM from MSCs and TE-MSCs, cells were cultured for 72 h in the presence or in the absence of NZ (10, 20, 30 μM) or ZA, then washed and incubated for additional 24 h in serum free (SF) medium. Conditioned medium (CM) from MSCs (MSCs-CM) and TE-MSCs (TE-MSCs-CM) were collected, filtered, aliquoted and stored at -20°C.

### ELISA assay

CCL5 (Pierce Biotecnology, Rockford, IL, USA), IL-6 (R&D System, Minneapolis, USA), IL-8 and VEGF (Immunological Sciences, Rome, Italy) and FGF-2 (PeproTech, Rocky Hill, NJ, USA) levels were assayed by ELISA assay, according with manufacturer's instructions.

### Proliferation and colony assay

MSCs (2×10^3^) were cultured in 96-well flat-bottomed plates, allowed to adhere for 24 h and then treated with NZ or ZA (15-30 μM) or blank nanoparticles (NPs). After 72 h, proliferation was evaluated by MTT assay. For clonogenic growth, 100 PC3 cells were plated in 24-well flat-bottomed plates, allowed to adhere for 24 hours, then cultured in the presence and in the absence of CM derived from tumor-educated MSCs (TE-MSCs) or MSCs cultured with or without NZ or ZA (20 μM). After 7 days, plates were observed under phase-contrast microscopy and colonies counted.

### MSCs differentiation

MSCs (1×10^4^) were cultured in 24-well plates and allowed to attach and then treated as described [12]. Briefly, at about 80% cell confluence, cells were pre-treated with 10 and 20 μM NZ or ZA for 6 h and then incubated, after a medium change, in osteogenic medium (OM) (50 nM ascorbic acid, 10 mM β-glycerol phosphate and 100 nM dexamethasone; Sigma-Aldrich) or in adipogenic medium (AM) (10 ng/mL Insulin, 1 μM dexamethasone, 60 nM indomethacin, and 0.5 mM isobutylmethyl-xanthyne; Sigma-Aldrich) for 3 weeks (pulse treatment). Images were taken using an inverted microscope (Eclipse TS/100; Nikon Instruments, Campi Bisenzio, Italy) with photomicrographic systems DS Camera Control Unit DS-L2 (magnification 100×0.25).

Osteogenic and adipocyte differentiation was assessed by Alizarin Red staining (Sigma-Aldrich) and with Oil Red-O staining (Sigma-Aldrich), respectively.

### Osteoclastogenic assay and TRAcP staining

Osteoclasts (OCs) were obtained as previously described [5, 59]. Peripheral blood mononuclear cells (PBMCs) were obtained from buffy coat preparations from healthy donors and separated on Ficoll-Hypaque (Pharmacia, Uppsala, Sweden). PBMC were seeded at optimal density, incubated for 2h at 37°C and then washed three times with PBS to remove non-adherent cells. Cultures were grown in RPMI supplemented with 10% FCS, human M-CSF (Peprotech, London) (30 ng/ml), and human RANKL (Peprotech) (40 ng/ml). Cells were fed every 3 days with fresh medium and differentiating factors in the absence and in the presence of increasing concentrations of NZ or ZA (25,50,100 nM). After 14 days of culture cells were used for tartrate-resistant acid phosphatase- (TRAcP) staining. To quantify the formation of TRAcP positive multinucleated cells, cell cultures were stained with Leukocyte Acid Phosphatase Kit (Sigma Diagnostics), according to manufacturer's instruction. Cells positive for TRAcP and having more than three nuclei were considered as TRAcP-positive multinucleated OCs.

### Cell motility assay

Migration was assessed by FATIMA assay as previously described [5, 60]. Briefly, MSCs (50.000 cells/insert) treated with NZ or ZA (10, 20, 30 μM), were tagged with the lipophylic dye Fast DiI (Molecular Probes) and then seeded in 150 μl SF medium in the upper side of fibronectin-coated Boyden chamber inserts. DMEM supplemented with 10% FBS was used as chemoattractant. In other experiments PC3 and DU145 cells (100.000 cells/inserts) were tagged with the lipophylic dye Fast DiI (Molecular Probes) and then seeded in 150 μl SF medium in the upper side of fibronectin-coated Boyden chamber inserts. Conditioned medium from MSCs treated or not with NZ or ZA were used as chemoattractants. Migration was monitored at different time intervals, using a computer-interfaced GeniusPlus microplate reader. FATIMA software determined the percentage of transmigrated cells out of the total amount introduced into the system.

### Software and statistical analysis of data

Values are presented as the mean with the standard deviation (mean ± SD) of three independent experiments. Statistical analysis was performed using GraphPad Prism 6 Software (GraphPad, San Diego, California, USA). The significance of differences was determined by Student's *t*-test for comparison between two groups. Analysis of Variances (ANOVA) was used to evaluate the correlation of data among three or more groups; consecutive multiple comparison analysis was performed using Dunnett's or Tukey's tests. *P*<0.05 was considered statistically significant. *P <0.05; **P <0.01; ***P <0.001; ****P <0.0001, treated *vs* control.

## CONCLUSIONS

ZA was recently used in association with Dasatinib for the treatment of Breast Cancer Bone Metastasis with good responses [57]. NZ, like ZA [13], inhibited the migration of MSCs and reduced the secretion of growth factors involved in breast cancer progression.

Therefore, since free ZA reaches only low intra-tumor concentrations, this new nanotechnology-based formulation, able to accumulate also in the extra skeletal tissues, might be able to kill cancer cells [22, 58] and block the cross-talk between MSCs and tumor cells, thus representing a valid alternative to ZA for the treatment of non-osseous metastases, not only in PCa but also in breast cancer.

## SUPPLEMENTARY FIGURE



## References

[1] Cammarota F, Laukkanen MO (2016). Mesenchymal Stem/Stromal Cells in Stromal Evolution and Cancer Progression. Stem Cells Int.

[2] Poggi A, Musso A, Dapino I, Zocchi MR (2014). Mechanisms of tumor escape from immune system: role of mesenchymal stromal cells. Immunol Lett.

[3] Sun Z, Wang S, Zhao RC (2014). The roles of mesenchymal stem cells in tumor inflammatory microenvironment. J Hematol Oncol.

[4] Bergfeld SA, Blavier L, DeClerck YA (2014). Bone marrow-derived mesenchymal stromal cells promote survival and drug resistance in tumor cells. Mol Cancer Ther.

[5] Borghese C, Cattaruzza L, Pivetta E, Normanno N, De Luca A, Mazzucato M, Celegato M, Colombatti A, Aldinucci D (2013). Gefitinib inhibits the cross-talk between mesenchymal stem cells and prostate cancer cells leading to tumor cell proliferation and inhibition of docetaxel activity. J Cell Biochem.

[6] Cheng J, Yang K, Zhang Q, Yu Y, Meng Q, Mo N, Zhou Y, Yi X, Ma C, Lei A, Liu Y (2016). The role of mesenchymal stem cells in promoting the transformation of androgen-dependent human prostate cancer cells into androgen-independent manner. Sci Rep.

[7] Yang X, Hou J, Han Z, Wang Y, Hao C, Wei L, Shi Y (2013). One cell, multiple roles: contribution of mesenchymal stem cells to tumor development in tumor microenvironment. Cell Biosci.

[8] Luo J, Lee SO, Cui Y, Yang R, Li L, Chang C (2015). Infiltrating bone marrow mesenchymal stem cells (BM-MSCs) increase prostate cancer cell invasion via altering the CCL5/HIF2α/androgen receptor signals. Oncotarget.

[9] Barcellos-de-Souza P, Comito G, Pons-Segura C, Taddei ML, Gori V, Becherucci V, Bambi F, Margheri F, Laurenzana A, Del Rosso M, Chiarugi P (2016). Mesenchymal Stem Cells are Recruited and Activated into Carcinoma-Associated Fibroblasts by Prostate Cancer Microenvironment-Derived TGF-β1. Stem Cells.

[10] Karlou M, Tzelepi V, Efstathiou E (2010). Therapeutic targeting of the prostate cancer microenvironment. Nat Rev Urol.

[11] Singh T, Kaur V, Kumar M, Kaur P, Murthy RS, Rawal RK (2015). The critical role of bisphosphonates to target bone cancer metastasis: an overview. J Drug Target.

[12] Ebert R, Zeck S, Krug R, Meissner-Weigl J, Schneider D, Seefried L, Eulert J, Jakob F (2009). Pulse treatment with zoledronic acid causes sustained commitment of bone marrow derived mesenchymal stem cells for osteogenic differentiation. Bone.

[13] Gallo M, De Luca A, Lamura L, Normanno N (2012). Zoledronic acid blocks the interaction between mesenchymal stem cells and breast cancer cells: implications for adjuvant therapy of breast cancer. Ann Oncol.

[14] Jia XH, Du Y, Mao D, Wang ZL, He ZQ, Qiu JD, Ma XB, Shang WT, Ding D, Tian J (2015). Zoledronic acid prevents the tumor-promoting effects of mesenchymal stem cells via MCP-1 dependent recruitment of macrophages. Oncotarget.

[15] Comito G, Pons Segura C, Taddei ML, Lanciotti M, Serni S, Morandi A, Chiarugi P, Giannoni E (2017). Zoledronic acid impairs stromal reactivity by inhibiting M2-macrophages polarization and prostate cancer-associated fibroblasts. Oncotarget.

[16] Clezardin P (2012). Potential anticancer properties of bisphosphonates: insights from preclinical studies. Anticancer Agents Med Chem.

[17] Vale CL, Burdett S, Rydzewska LH, Albiges L, Clarke NW, Fisher D, Fizazi K, Gravis G, James ND, Mason MD, Parmar MK, Sweeney CJ, Sydes MR, STOpCaP Steering Group (2016). Addition of docetaxel or bisphosphonates to standard of care in men with localised or metastatic, hormone-sensitive prostate cancer: a systematic review and meta-analyses of aggregate data. Lancet Oncol.

[18] Pattni BS, Chupin VV, Torchilin VP (2015). New Developments in Liposomal Drug Delivery. Chem Rev.

[19] Bobo D, Robinson KJ, Islam J, Thurecht KJ, Corrie SR (2016). Nanoparticle-Based Medicines: A Review of FDA-Approved Materials and Clinical Trials to Date. Pharm Res.

[20] Xing H, Hwang K, Lu Y (2016). Recent Developments of Liposomes as Nanocarriers for Theranostic Applications. Theranostics.

[21] Salzano G, Marra M, Porru M, Zappavigna S, Abbruzzese A, La Rotonda MI, Leonetti C, Caraglia M, De Rosa G (2011). Self-assembly nanoparticles for the delivery of bisphosphonates into tumors. Int J Pharm.

[22] Marra M, Salzano G, Leonetti C, Tassone P, Scarsella M, Zappavigna S, Calimeri T, Franco R, Liguori G, Cigliana G, Ascani R, La Rotonda MI, Abbruzzese A (2011). Nanotechnologies to use bisphosphonates as potent anticancer agents: the effects of zoledronic acid encapsulated into liposomes. Nanomedicine (Lond).

[23] Kopecka J, Porto S, Lusa S, Gazzano E, Salzano G, Giordano A, Desiderio V, Ghigo D, Caraglia M, De Rosa G, Riganti C (2015). Self-assembling nanoparticles encapsulating zoledronic acid revert multidrug resistance in cancer cells. Oncotarget.

[24] Kopecka J, Porto S, Lusa S, Gazzano E, Salzano G, Pinzòn-Daza ML, Giordano A, Desiderio V, Ghigo D, De Rosa G, Caraglia M, Riganti C (2016). Zoledronic acid-encapsulating self-assembling nanoparticles and doxorubicin: a combinatorial approach to overcome simultaneously chemoresistance and immunoresistance in breast tumors. Oncotarget.

[25] Mognetti B, La Montagna G, Perrelli MG, Pagliaro P, Penna C (2013). Bone marrow mesenchymal stem cells increase motility of prostate cancer cells via production of stromal cell-derived factor-1α. J Cell Mol Med.

[26] Aldinucci D, Celegato M, Casagrande N (2016). Microenvironmental interactions in classical Hodgkin lymphoma and their role in promoting tumor growth, immune escape and drug resistance. Cancer Lett.

[27] Houthuijzen JM, Daenen LG, Roodhart JM, Voest EE (2012). The role of mesenchymal stem cells in anti-cancer drug resistance and tumour progression. Br J Cancer.

[28] Katz OB, Shaked Y (2015). Host effects contributing to cancer therapy resistance. Drug Resist Updat.

[29] Chang AI, Schwertschkow AH, Nolta JA, Wu J (2015). Involvement of mesenchymal stem cells in cancer progression and metastases. Curr Cancer Drug Targets.

[30] Barcellos-de-Souza P, Gori V, Bambi F, Chiarugi P (2013). Tumor microenvironment: bone marrow-mesenchymal stem cells as key players. Biochim Biophys Acta.

[31] Laurent V, Guérard A, Mazerolles C, Le Gonidec S, Toulet A, Nieto L, Zaidi F, Majed B, Garandeau D, Socrier Y, Golzio M, Cadoudal T, Chaoui K (2016). Periprostatic adipocytes act as a driving force for prostate cancer progression in obesity. Nat Commun.

[32] Shiao SL, Chu GC, Chung LW (2016). Regulation of prostate cancer progression by the tumor microenvironment. Cancer Lett.

[33] Wang N, Docherty FE, Brown HK, Reeves KJ, Fowles AC, Ottewell PD, Dear TN, Holen I, Croucher PI, Eaton CL (2014). Prostate cancer cells preferentially home to osteoblast-rich areas in the early stages of bone metastasis: evidence from in vivo models. J Bone Miner Res.

[34] Haider MT, Holen I, Dear TN, Hunter K, Brown HK (2014). Modifying the osteoblastic niche with zoledronic acid in vivo-potential implications for breast cancer bone metastasis. Bone.

[35] Herroon MK, Rajagurubandara E, Hardaway AL, Powell K, Turchick A, Feldmann D, Podgorski I (2013). Bone marrow adipocytes promote tumor growth in bone via FABP4-dependent mechanisms. Oncotarget.

[36] Lapeire L, Hendrix A, Lambein K, Van Bockstal M, Braems G, Van Den Broecke R, Limame R, Mestdagh P, Vandesompele J, Vanhove C, Maynard D, Lehuédé C, Muller C (2014). Cancer-associated adipose tissue promotes breast cancer progression by paracrine oncostatin M and Jak/STAT3 signaling. Cancer Res.

[37] Hensel J, Thalmann GN (2016). Biology of Bone Metastases in Prostate Cancer. Urology.

[38] Diedrich JD, Rajagurubandara E, Herroon MK, Mahapatra G, Hüttemann M, Podgorski I (2016). Bone marrow adipocytes promote the Warburg phenotype in metastatic prostate tumors via HIF-1α activation. Oncotarget.

[39] Hefetz-Sela S, Scherer PE (2013). Adipocytes: impact on tumor growth and potential sites for therapeutic intervention. Pharmacol Ther.

[40] Mathew A, Brufsky A (2014). Breast cancer: zoledronic acid—more than just a bone drug. Nat Rev Clin Oncol.

[41] Hardaway AL, Herroon MK, Rajagurubandara E, Podgorski I (2014). Bone marrow fat: linking adipocyte-induced inflammation with skeletal metastases. Cancer Metastasis Rev.

[42] Kamba T, Kamoto T, Maruo S, Kikuchi T, Shimizu Y, Namiki S, Fujimoto K, Kawanishi H, Sato F, Narita S, Satoh T, Saito H, Sugimoto M, ZAPCA Study Group (2017). A phase III multicenter, randomized, controlled study of combined androgen blockade with versus without zoledronic acid in prostate cancer patients with metastatic bone disease: results of the ZAPCA trial. Int J Clin Oncol.

[43] Marra M, Salzano G, Leonetti C, Porru M, Franco R, Zappavigna S, Liguori G, Botti G, Chieffi P, Lamberti M, Vitale G, Abbruzzese A, La Rotonda MI (2012). New self-assembly nanoparticles and stealth liposomes for the delivery of zoledronic acid: a comparative study. Biotechnol Adv.

[44] Caraglia M, Marra M, Naviglio S, Botti G, Addeo R, Abbruzzese A (2010). Zoledronic acid: an unending tale for an antiresorptive agent. Expert Opin Pharmacother.

[45] Yao X, Huang J, Zhong H, Shen N, Faggioni R, Fung M, Yao Y (2014). Targeting interleukin-6 in inflammatory autoimmune diseases and cancers. Pharmacol Ther.

[46] Gu L, Talati P, Vogiatzi P, Romero-Weaver AL, Abdulghani J, Liao Z, Leiby B, Hoang DT, Mirtti T, Alanen K, Zinda M, Huszar D, Nevalainen MT (2014). Pharmacologic suppression of JAK1/2 by JAK1/2 inhibitor AZD1480 potently inhibits IL-6-induced experimental prostate cancer metastases formation. Mol Cancer Ther.

[47] Vaday GG, Peehl DM, Kadam PA, Lawrence DM (2006). Expression of CCL5 (RANTES) and CCR5 in prostate cancer. Prostate.

[48] Luo J, Ok Lee S, Liang L, Huang CK, Li L, Wen S, Chang C (2014). Infiltrating bone marrow mesenchymal stem cells increase prostate cancer stem cell population and metastatic ability via secreting cytokines to suppress androgen receptor signaling. Oncogene.

[49] Velasco-Velázquez M, Xolalpa W, Pestell RG (2014). The potential to target CCL5/CCR5 in breast cancer. Expert Opin Ther Targets.

[50] Kato T, Fujita Y, Nakane K, Mizutani K, Terazawa R, Ehara H, Kanimoto Y, Kojima T, Nozawa Y, Deguchi T, Ito M (2013). CCR1/CCL5 interaction promotes invasion of taxane-resistant PC3 prostate cancer cells by increasing secretion of MMPs 2/9 and by activating ERK and Rac signaling. Cytokine.

[51] Aldinucci D, Colombatti A (2014). The inflammatory chemokine CCL5 and cancer progression. Mediators Inflamm.

[52] Velasco-Velázquez M, Pestell RG (2013). The CCL5/CCR5 axis promotes metastasis in basal breast cancer. OncoImmunology.

[53] Karnoub AE, Dash AB, Vo AP, Sullivan A, Brooks MW, Bell GW, Richardson AL, Polyak K, Tubo R, Weinberg RA (2007). Mesenchymal stem cells within tumour stroma promote breast cancer metastasis. Nature.

[54] Yu PF, Huang Y, Han YY, Lin LY, Sun WH, Rabson AB, Wang Y, Shi YF (2017). TNFα-activated mesenchymal stromal cells promote breast cancer metastasis by recruiting CXCR2(+) neutrophils. Oncogene.

[55] Mittal K, Ebos J, Rini B (2014). Angiogenesis and the tumor microenvironment: vascular endothelial growth factor and beyond. Semin Oncol.

[56] Finley SD, Chu LH, Popel AS (2015). Computational systems biology approaches to anti-angiogenic cancer therapeutics. Drug Discov Today.

[57] Mitri Z, Nanda R, Blackwell K, Costelloe CM, Hood I, Wei C, Brewster AM, Ibrahim NK, Koenig KB, Hortobagyi GN, Van Poznak C, Rimawi MF, Moulder-Thompson S, Translational Breast Cancer Research Consortium (2016). TBCRC-010: Phase I/II Study of Dasatinib in Combination with Zoledronic Acid for the Treatment of Breast Cancer Bone Metastasis. Clin Cancer Res.

[58] Caraglia M, Luongo L, Salzano G, Zappavigna S, Marra M, Guida F, Lusa S, Giordano C, De Novellis V, Rossi F, Abbruzzese Saccardi A, De Rosa G, Maione S (2013). Stealth liposomes encapsulating zoledronic acid: a new opportunity to treat neuropathic pain. Mol Pharm.

[59] Pivetta E, Scapolan M, Wassermann B, Steffan A, Colombatti A, Spessotto P (2011). Blood-derived human osteoclast resorption activity is impaired by Hyaluronan-CD44 engagement via a p38-dependent mechanism. J Cell Physiol.

[60] Spessotto P, Lacrima K, Nicolosi PA, Pivetta E, Scapolan M, Perris R (2009). Fluorescence-based assays for in vitro analysis of cell adhesion and migration. Methods Mol Biol.

