# Supplementary material for: Self-assembling nanoparticles encapsulating zoledronic acid inhibit mesenchymal stromal cells differentiation, migration and secretion of proangiogenic factors and their interactions with prostate cancer cells
Source: Oncotarget. 2017 Apr 19;8(26):42926–38. doi: 10.18632/oncotarget.17216 (PMC5522116; doi:10.18632/oncotarget.17216)
Supplement: Supplementary file 1 [file oncotarget-08-42926-s001.pdf]

Self-assembling nanoparticles encapsulating zoledronic acid inhibit mesenchymal stromal cells differentiation, migration and secretion of proangiogenic factors and their interactions with prostate cancer cells

SUPPLEMENTARY FIGURE

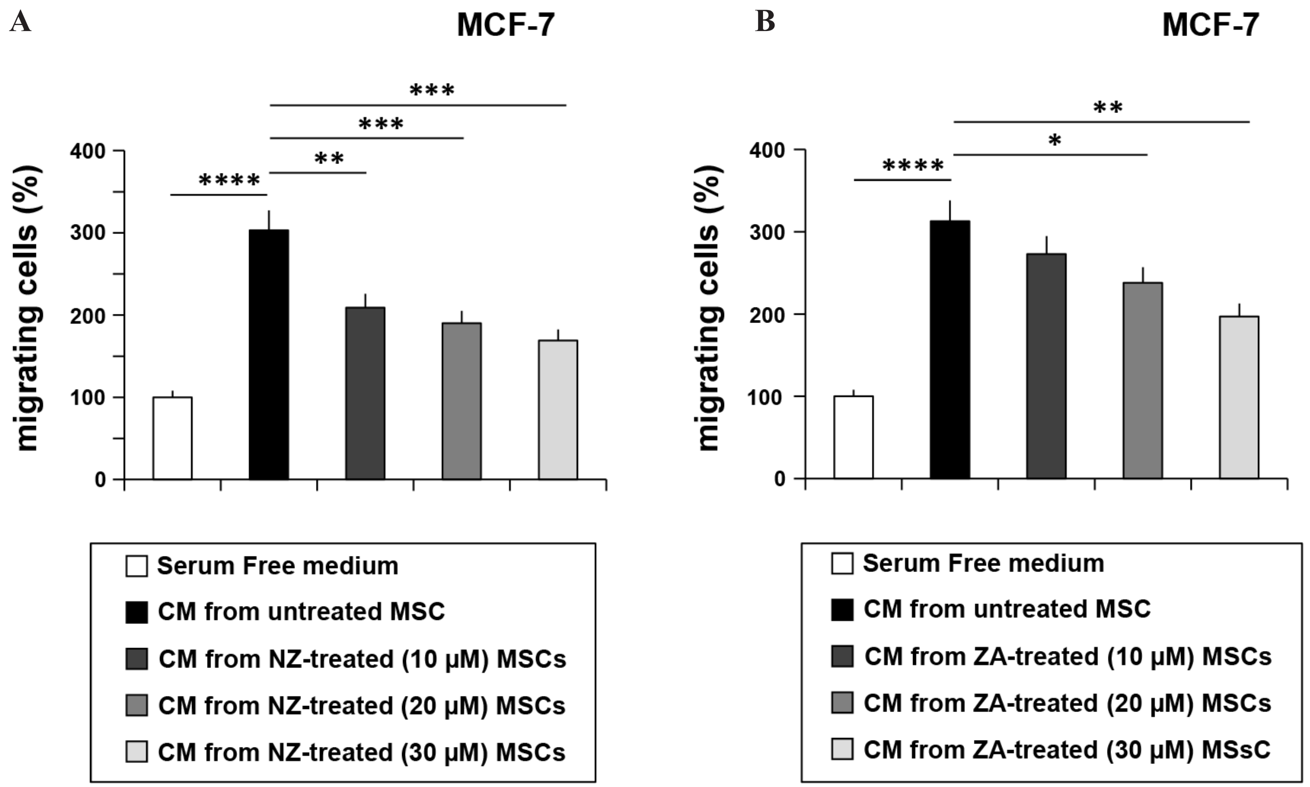

Supplementary Figure 1: Treatment of MSCs with NZ or ZA decreased the migration of breast cancer cells induced by MSCs-CM. Migration of MCF-7 cells through a fibronectin-coated Boyden chamber in response to serum free medium (control=100%), CM from MSCs untreated or treated with **(A)** NZ or **(B)** ZA. Histograms represent the percentage of transmigrated cells after 20 h relative to control (cells migrated towards serum free medium). Values represent the mean ± SD of three different experiments.
